# Progress in Diseases of the Blood

**Published:** 1902-11-01

**Authors:** 


### PROGRESS IN DISEASES OF THE BLOOD.

**Splenic Anæmia.**—Rolleston[1] discusses two cases under his care, in one of which splenectomy was done, but the patient died from hæmatemesis. The spleen weighed 37 ounces, and there was no enlargement of the liver, jaundice or ascites, the blood showed red cells reduced by one-half, and the hæmoglobin by three-quarters. The white cells were only 3,500, a curious scarcity of leucocytes which occurs in some cases of this disease. The second patient, though at first it appeared a normal instance, developed enlarged glands in the axilla and groin, and reacted to tuberculin. Hence it was probably tuberculosis of the spleen and glands, if not lymphadenoma. An interesting symptom was albuminuria, which disappeared when the patient walked about, and returned when she was in bed. This may have been due to the pressure of the spleen on the left renal vein. Pathologically he regards splenic anæmia as a lymphadenoma of the spleen, which remains localised, possibly because the liver lies between it and the general circulation. Moreover, as splenectomy has been known to cure the disease there can be no generalised infection, and for the same reason it cannot be due to a deficient action of the spleen. The cirrhosis of the liver and ascites which may occur in the later stages, and has been called Banti's disease, is consistent with such a formation of a toxin in the spleen. The endothelial proliferation which is found here shows some analogy to tuberculous adenitis. Ewing, too, notes that the blood changes are the same as those found in gumma and lymphadenoma of the spleen. The results of splenectomy are good, 14 out of 18 cases recovered after the operation, and an increase of eosinophile cells is usually noticed. Dr. Murrell[2] gives references to a number of records of Banti's disease, and reports an acute case in a woman, aged 31, whose illness commenced with hæmatemesis and fever, and lasted only 16 days. The red cells never exceeded 1,080,000, the white cells 4,375, and the hæmoglobin 20 per cent. The liver weighed 50 ounces and the spleen 9, and showed some cirrhosis. The question arises was this pernicious anæmia or Banti's disease? It certainly does not agree with Dreschfield and Rolleston's theory that the latter is a final stage of splenic anæmia, but Murrell would class it under that name, and quotes Cabot as including under that head cases where the red cells are more reduced than the hæmoglobin. The differential count in his own case seems insufficient to settle the question of pernicious anæmia. Some forty cases only of splenic anæmia are on record. Beverley Robinson[3] gives an instance of the good results of splenectomy in a boy of 14 suffering from this disease. The red cells numbered 4,600,000 and the hæmoglobin 77 per cent. The spleen was removed eight years ago, and the young man is now in excellent health. After the operation leucocytosis occurred, but the blood finally became normal. Bryant, however, thinks the operation mortality in pseudo-leukæmia is 76 per cent., and in true leukæmia over 90 per cent. J. W. Brannan in the same discussion referred to another patient, aged 13, who was treated with iodide of iron, arsenic, and saline rectal injections with remarkable improvement, the red cells rising from 1,600,000 to 4,350,000, the hæmoglobin from 18 to 75 per cent., and the spleen diminishing. Dreschfeld,[4] showing a case of Banti's disease, remarked on the large number of lymphocytes, though the total leucocytes were diminished. This he thinks may be of use in diagnosis. Besides the cases of splenic anæmia which are really the first stage of Banti's disease, he refers to those due to malaria, infantile forms, those which are due to Hodgkin's disease, and those few cases of the pure type in which the blood shows the signs of chlorosis.

**Leukæmia.**—G. Parker[5] reported the first instance in this country of the combination of splenic leukæmia and phthisis. For some reason these diseases hardly ever occur together. Whether this is due to the protective power of the xanthin bases from leucocytosis in the former disease or no, only two clear instances have been hitherto noticed, and both were in America. This patient, a male aged 34, had suffered from phthisis of old standing, with great flattening of the chest wall and laryngeal complication, but had regained vigorous health and strength, when he fell and struck his side, and an enlargement of the spleen followed. Eighteen months later the white cells of the blood reached 238,000, and then 640,000, with a large number of myelocytes, eosinophiles, and some normoblasts. The lung disease progressed, and the white cells fell to 100,000, but this may have been due to the large doses of cacodylate of soda he took. In lymphatic leukæmia the complication is less rare, and may be accompanied with a fall in the leucocytes, such as is produced by occurrence of a septic or other infection during the course of leukæmia. W. Murrell[6] has since found a similar case in a woman, aged 21, in whom the leucocytes reached 220,000, with many myelocytes and eosinophiles. There were hardly any lung symptoms during life, but afterwards the lungs showed marked tubercular consolidation, and in the enlarged spleen tubercles also existed. He refers to the ulcerative process found in the lungs of some leukæmic patients, with the formation of lymphatic nodules. These have been chiefly observed in lymphatic leukæmia, and may be easily mistaken for tubercle. A case showing the difficulty of diagnosis is given by D. K. Williamson and E. W. Martin.[7] The red cells sank to 300,000, the white were increased to 45,000, with no less than 99 per cent. of lymphocytes. The hæmoglobin was reduced to about the extent of the red cells, and marked poikilocytosis was present. Thus it might be regarded either as pernicious anæmia or lymphatic leukæmia, and the course was rapidly fatal though there were no hæmorrhages on the skin or mucous membranes. With regard to the origin of the normal blood cells, R. Muir[8] shows that the polymorphonuclear ones are derived from myelocytes in the marrow, and that their production is greatly increased during inflammatory conditions. In small-pox, where a great diminution of the cells occurs, degenerative changes, due to a toxin, are found in the marrow itself.

[1] Clin. Jour., April 16. [2] Lancet, April 26. [3] Med. Rec., Jan. 18. [4] Brit. Med. Jour., March 24. [5] Brit. Med. Jour., May 10. [6] Lancet, July 19. [7] Brit. Med. Jour., May 10. [8] Brit. Med. Jour., Feb. 22.

*(To be continued.)*

